# WY-14643 Regulates CYP1B1 Expression through Peroxisome Proliferator-Activated Receptor α-Mediated Signaling in Human Breast Cancer Cells

**DOI:** 10.3390/ijms20235928

**Published:** 2019-11-25

**Authors:** Yong Pil Hwang, Seong Su Won, Sun Woo Jin, Gi Ho Lee, Thi Hoa Pham, Jae Ho Choi, Keon Wook Kang, Hye Gwang Jeong

**Affiliations:** 1 Department of Pharmaceutical Engineering, International University of Korea, Jinju 52833, Korea; protoplast@hanmail.net; 2 College of Pharmacy, Chungnam National University, Daejeon 34134, Korea; blueberry8686@daum.net (S.S.W.); mpassword@cnu.ac.kr (S.W.J.); ghk1900@cnu.ac.kr (G.H.L.); hoapt@cnu.ac.kr (T.H.P.); choijh1@cnu.ac.kr (J.H.C.); 3 College of Pharmacy and Research Institute of Pharmaceutical Sciences, Seoul National University, Seoul 08826, Korea; kwkang@snu.ac.kr

**Keywords:** CYP1B1, PPRE, PPARα, WY-14643, human breast carcinoma MCF-7

## Abstract

Human cytochrome P450 1B1 (CYP1B1)-mediated biotransformation of endobiotics and xenobiotics plays an important role in the progression of human breast cancer. In this study, we investigated the effects of WY-14643, a peroxisome proliferator-activated receptor α (PPARα) agonist, on CYP1B1 expression and the related mechanism in MCF7 breast cancer cells. We performed quantitative reverse transcription-polymerase chain reaction, transient transfection, and chromatin immunoprecipitation to evaluate the effects of PPARα on peroxisome proliferator response element (PPRE)-mediated transcription. WY-14643 increased the protein and mRNA levels of CYP1B1, as well as promoter activity, in MCF-7 cells. Moreover, WY-14643 plus GW6471, a PPARα antagonist, significantly inhibited the WY-14643-mediated increase in CYP1B1 expression. PPARα knockdown by a small interfering RNA markedly suppressed the induction of CYP1B1 expression by WY-14643, suggesting that WY-14643 induces CYP1B1 expression via a PPARα-dependent mechanism. Bioinformatics analysis identified putative PPREs (−833/−813) within the promoter region of the CYP1B1 gene. Inactivation of these putative PPREs by deletion mutagenesis suppressed the WY-14643-mediated induction of CYP1B1 promoter activation. Furthermore, WY-14643 induced PPARα to assume a form capable of binding specifically to the PPRE-binding site in the CYP1B1 promoter. Our findings suggest that WY-14643 induces the expression of CYP1B1 through activation of PPARα.

## 1. Introduction

Breast cancer is the most common cancer in women and the second most common cause of cancer-related death in women worldwide [1]. Estrogens are related to the risk of development of human breast cancer [2]. Cytochromes P450 (CYPs) are hemoproteins involved in the metabolism and degradation of several endogenous compounds, including estradiol and fatty acids. They are also involved in the bioactivation of environmental procarcinogens, such as arylamines and polycyclic aromatic hydrocarbons. Among the CYPs, CYP1A1 and CYP1B1 play physiological roles in the degradation of 17 β-estradiol (E2; the main estrogen) into non-carcinogenic 2-hydroxyestradiol and carcinogenic 4-hydroxyestradiol (4-OHE2) in breast tissues and mammary glands [2,3]. CYP1B1 is presumed to be essential for the initiation and development of various hormone-dependent tumors, including breast cancer, through the biotransformation of endogenous estrogens and environmental carcinogens [4]. Indeed, elevated and consistent expression of CYP1B1 has been confirmed in breast tumors [5]. The 17-β-estradiol-3,4-quinones generated by oxidation of 4-hydroxyestradiol produce predominantly mutagenic DNA adducts and reactive oxygen species, which cause oxidative damage to the cell and lead to tumor initiation [6]. In contrast, the 17-β-estradiol-2,3-semiquinones derived from 2-hydroxyestradiol produce stable and less harmful DNA adducts [6]. Based on these observations, inhibition of CYP1B1, an important drug target, has been proposed as a therapeutic strategy for breast cancer [5].

CYP1B1 is regulated by estrogen receptor, aryl hydrocarbon receptor (AhR), aryl hydrocarbon receptor nuclear translocator complex, cyclic AMP–response element–binding protein, and the Sp1 ligand-activated transcription factor [7,8]. In addition, disruption of the *Cyp1b1* gene has been shown to alter the expression levels of 560 liver genes, including suppression of peroxisome proliferator-activated receptor (PPAR) γ and multiple genes regulated by PPARα [9]. However, the activation of transcription factors does not always explain the induction of CYP1B1 expression. PPARs are ligand-dependent transcription factors [10,11] that are involved in the regulation of glucose homeostasis, lipid metabolism, inflammation, proliferation, cell cycle, differentiation, and cell death [9,12]. PPARs share a highly conserved structure and molecular mode of action as a heterodimer with the retinoid X receptor (RXR), recognizing specific DNA sequences in target genes known as peroxisome proliferator response elements (PPREs) [13,14]. The three PPAR subtypes (PPARα, PPARγ, and PPAR β/δ) are often activated in tumors, where they modulate cell survival, differentiation, and proliferation, critical aspects of cancer biology [15]. PPARα is important in several malignant tumors, including breast cancer [16], hepatocellular carcinoma [17], chronic lymphocytic leukemia [18], glioblastoma [19,20], and renal cancer [21]. PPARα-deficient mice were reportedly refractory to the liver-carcinogenic effect of the PPARα agonist WY-14643 [22]. In addition, the growth and progression of lung carcinoma and melanoma engrafted in wild-type mice were completely suppressed when these tumors were implanted in PPARα-deficient mice [23]. Intriguingly, PPAR*α*, which is activated by the chemical agonist WY-14643, induces both pro- and anti-inflammatory responses in target cells [16,24,25]. Therefore, PPARα is a promising target for the treatment of cancer.

In this work, we demonstrate that the expression of CYP1B1 is highly induced by the PPARα agonist, WY-14643, in a manner involving the transcription factor PPARα and requiring PPRE sites located within the CYP1B1 promoter.

## 2. Results

### 2.1. Cytotoxicity of WY-14643 in MCF-7 Breast Cancer Cells

**Figure 1:**
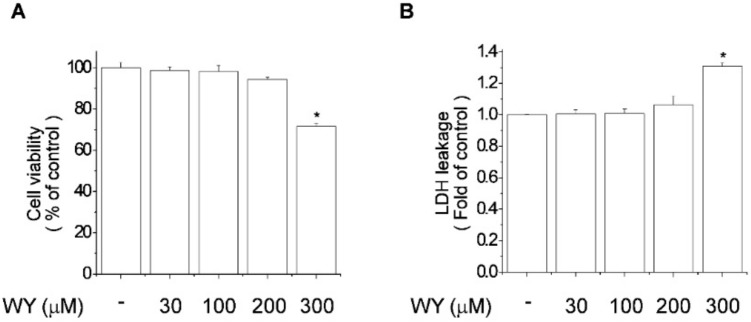
Effect of WY-14643 (0, 30 100, 200, or 300 μM; 24 h) on the viability of MCF-7 cells. Cell viability and lactate dehydrogenase release were determined by (**A**) MTT and (**B**) lactate dehydrogenase release assays, respectively. Bars are means ± standard deviations of three independent experiments performed in triplicate. * *p* < 0.01, significantly different from the control.

The toxicity of WY-14643 to MCF-7 breast cancer cells was evaluated by MTT (Figure 1A) and lactate dehydrogenase (Figure 1B) assays. MCF-7 cells were exposed to WY-14643 (30, 100, 200, or 300 μM) for 24 h. WY-14643 at < 200 μM did not exert a cytotoxic effect, while WY-14643 at 300 μM resulted in a 28% reduction in cell viability (Figure 1). Thus, WY-14643 at < 200 μM did not exert a significant cytotoxic effect on MCF-7 cells.

### 2.2. WY-14643 Induced CYP1B1 Expression, Activity, and Promoter Activity in MCF-7 Cells

**Figure 2:**
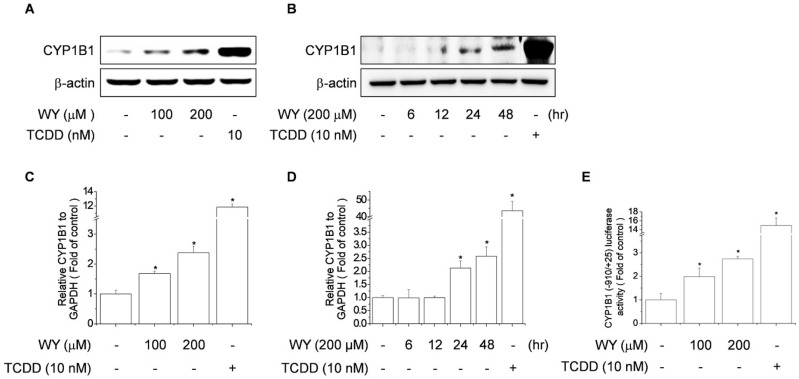
Effect of WY-14643 on CYP1B1 expression in MCF-7 cells. (**A**) Effect of WY-14643 on CYP1B1 protein levels. Cells were treated with WY-14643 (100–200 μM) or TCDD (10 nM) for 24 h and CYP1B1 protein levels were analyzed by immunoblotting of cell lysates using an anti-hCYP1B1 antibody. (**B**) Cells were cultured with 200 μM WY-14643 for 6, 12, 24, or 48 h and CYP1B1 protein levels were analyzed by immunoblotting of cell lysates using an anti-hCYP1B1 antibody. (**C**) Effect of WY-14643 on CYP1B1 mRNA levels. Cells were treated with WY-14643 (100–200 μM) or TCDD (10 nM) for 24 h, then lysed; total RNA was prepared for PCR analysis of CYP1B1 mRNA levels, relative to the level of GAPDH. (**D**) Cells were cultured with 200 μM WY-14643 for 6, 12, 24, or 48 h or TCDD (10 nM) for 48 h, then lysed; total RNA was prepared for PCR analysis of CYP1B1 mRNA levels, relative to the level of GAPDH. (**E**) Effect of WY-14643 on CYP1B1 promoter activity. Cells transfected with CYP1B1-Luc (−910/+25) were treated with WY-14643 (100–200 μM) or TCDD (10 nM) for 24 h. Cells were harvested and assayed for luciferase activity, which was normalized to the activity of β-galactosidase. Bars are means ± standard deviations of three independent experiments performed in triplicate. * *p* < 0.01, significantly different from the control.

To examine the effect of WY-14643 on CYP1B1 expression, MCF-7 human breast carcinoma cells were treated with WY-14643 (100 or 200 μM) or TCDD (10 nM) for 24 h and CYP1B1 protein levels were assayed by immunoblotting. TCDD was used as the positive control instead of WY-14643 because it activates aryl hydrocarbon receptor and the expression of its target genes [26], such as CYP1B1. MCF-7 cells exposed to WY-14643 for 24 h showed a concentration-dependent increase in the protein and mRNA levels of CYP1B1 (Figure 2A,C). Treatment with 200 μM WY-14643 resulted in a time-dependent increase in the protein and mRNA levels of CYP1B1 (Figure 2B,D). These results implicate WY-14643 in the transcriptional activation of CYP1B1 in MCF-7 cells. To investigate the mechanism by which WY-14643 regulates CYP1B1 expression, MCF-7 cells were transfected with the CYP1B1-Luc reporter construct; this revealed that WY-14643 induced CYP1B1 luciferase activity in MCF-7 cells (Figure 2E). We also measured the expression of CYP1B1 gene in another breast cancer cell line, MDA-MB-231 by WY1234 treatment. As shown in Figure S1, WY-14643 induced the mRNA levels of CYP1B1 in MDA-MB231 cells in a concentration-dependent manner (Figure S1). To achieve the solid results, we measured the expression of CYP1B1 by using another PPARα agonist, fenofibrate. As shown in Figure S2, fenofibrate induced the mRNA levels of CYP1B1 in MCF-7 cells in a concentration-dependent manner (Figure S2).

### 2.3. WY-14643 Induces CYP1B1 Expression in MCF-7 Cells Via a PPARα-Dependent Mechanism

**Figure 3:**
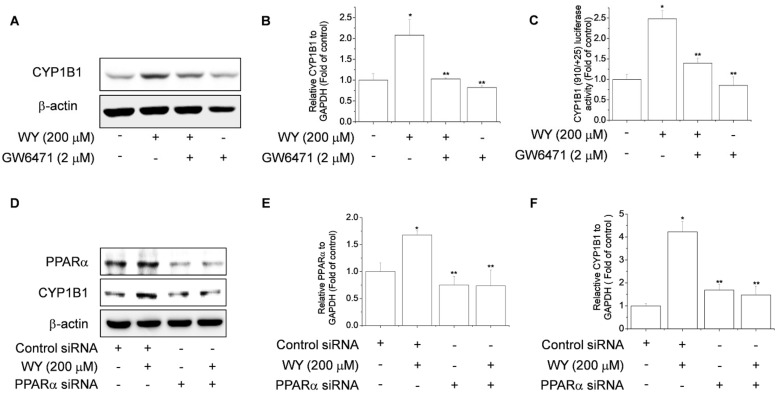
WY-14643 induces CYP1B1 expression via a PPARα-dependent mechanism in MCF-7 cells. (**A**) Effect of PPARα on the WY-14643-induced increase in CYP1B1 protein levels in MCF-7 cells. Cells were pretreated with 2 μM GW6471, an antagonist of PPARα, and incubated with 200 μM WY-14643 for 24 h. CYP1B1 protein levels were analyzed by immunoblotting of cell lysates using an anti-hCYP1B1 antibody. (**B**) Effect of PPARα on the WY-14643-induced increase in CYP1B1 mRNA levels. Cells were pretreated with 2 μM GW6471, an antagonist of PPARα, and incubated with 200 μM WY-14643 for 24 h. Cells were lysed and total RNA was prepared for PCR analysis of CYP1B1 mRNA levels, relative to the level of GAPDH. (**C**) Effect of PPARα on WY-14643-induced CYP1B1 promoter activity. Cells were pretreated with 2 μM GW6471, an antagonist of PPARα, and incubated with 200 μM WY-14643 for 24 h. Cells were harvested and assayed for luciferase activity, which was normalized to the activity of β-galactosidase. (**D**) Cells were transfected with PPARα siRNA or a nonspecific control siRNA, in accordance with the manufacturer’s instructions. After 48 h, cells were treated with 200 μM WY-14643 for 24 h and the PPARα and CYP1B1 protein levels were analyzed by immunoblotting using anti-PPARα and -hCYP1B1 antibodies. (**E**,**F**) MCF-7 cells were transfected with PPARα siRNA or a nonspecific control siRNA, in accordance with the manufacturer’s instructions. After 48 h, cells were treated with 200 μM WY-14643 for 24 h. Cells were lysed and total RNA was prepared for PCR analysis of the PPARα and CYP1B1 mRNA levels, relative to the level of GAPDH. Bars are means ± standard deviations of three independent experiments performed in triplicate. * *p* < 0.01, significantly different from the control. ** *p* < 0.01, significantly different from the WY-14643 treatment.

To examine whether the induction of CYP1B1 by WY-14643 is mediated by a PPARα-dependent pathway, we investigated the effect of GW6471, a PPARα antagonist, on WY-14643-induced expression of CYP1B1. WY-14643 increased the CYP1B1 mRNA and protein levels, as well as luciferase activity, in MCF-7 cells (Figure 3A–C). However, treatment with WY-14643 plus GW6471 markedly suppressed the CYP1B1 mRNA and protein levels, as well as luciferase activity, compared to WY-14643 alone (Figure 3A–C). Next, we used a PPARα siRNA to confirm the effect of PPARα on CYP1B1. The PPARα siRNA significantly reduced the PPARα mRNA and protein levels, compared to the control siRNA (Figure 3D,E). PPARα knockdown significantly suppressed the WY-14643-induced increases in CYP1B1 mRNA and protein levels (Figure 3D,F). These findings indicated that WY-14643 induces CYP1B1 expression via a PPARα-dependent mechanism in breast cancer cells.

### 2.4. WY-14643 Upregulates CYP1B1 Expression by Activating the PPRE-Binding Site

**Figure 4:**
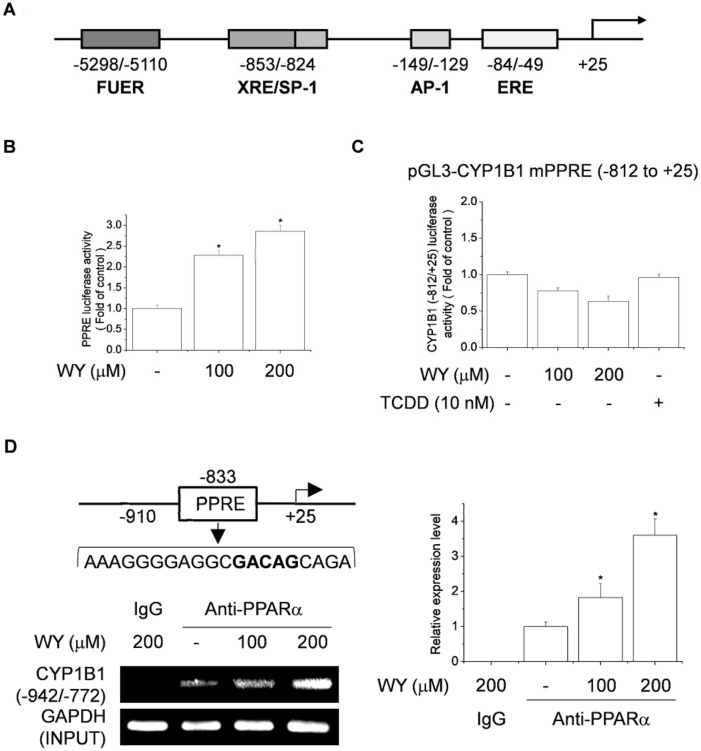
WY-14643 induces CYP1B1 expression by activating the PPRE-binding site in MCF-7 cells. (**A**) Schematic of hCYP1B1 promoter. (**B**) Effect of WY-14643 on PPRE activity. Cells were transfected with 1 μg of PPRE-Luc vector and 0.2 μg of pCMV-β-gal, then treated with WY-14643 (100–200 μM) for 24 h. Cells were harvested and assayed for luciferase activity, which was normalized to the activity of β-galactosidase. (**C**) Effect of WY-14643 on the activity of CYP1B1 promoter regions. Cells were transfected with 1 μg of hCYP1B1 promoter deletion construct (−812/+25) and 0.2 μg of pCMV-β-gal for 4 h, then treated with WY-14643 (100–200 μM) or TCDD (10 nM) for 24 h. Cells were harvested and assayed for luciferase activity, which was normalized to the activity of β-galactosidase. (**D**) Effect of WY-14643 on binding of PPARα to the CYP1B1 site in MCF-7 cells. Cells were treated with 200 μM WY-14643 for 24 h. Using an anti-PPARα antibody, ChIP was performed on chromatin extracted from WY-14643-stimulated cells, and CYP1B1 regions were amplified by PCR. The products were electrophoresed in a 2% agarose gel and stained with SYBR Green. Bars are means ± standard deviations of three independent experiments performed in triplicate. * *p* < 0.01, significantly different from the control.

The ERE (−84/49), activator protein 1 (−149/−129), and SP-1/XRE (−853/−824) transcription factor-binding sites in the human CYP1B1 promoter (Figure 4A) are important for regulating the transcription of CYP1B1 in cancer cells [7,27]. To investigate whether WY-14643 contributes to the effect of PPARα on the CYP1B1 promoter, MATCH software was used to analyze the human CYP1B1 promoter sequence. This software is designed to search for potential transcription factor-binding sites in the nucleotide sequence. The results showed a putative PPAR/RXR-binding site starting at position −833, relative to the transcription start site. MCF-7 cells then transfected with a PPRE-luciferase vector and the cells were stimulated with WY-14643 for 24 h. WY-14643 significantly induced PPRE-luciferase activity in a concentration-dependent manner (Figure 4B). To identify the region of the CYP1B1 promoter that mediates the inductive effect of WY-14643, cells were transfected with the −812/+25 CYP1B1 deletion construct, which excludes PPRE transcription factor-binding sites.

The −812/+25 CYP1B1 deletion construct contains activator protein 1 and ERE transcription factor-binding sites. Transcriptional activation by WY-14643 was not detected in the promoter of the −812/+25 CYP1B1 deletion mutant (Figure 4C). Therefore, the PPRE region is not located in the −812/+25 region of the CYP1B1 promoter. To examine the binding of PPARα to the CYP1B1 promoter, performed ChIP assays and qPCR were performed using specific primers to amplify 833-bp regions that included putative PPRE motifs at −942 to −772 bp upstream of the transcription start site (Figure 4D). The binding of PPARα to the CYP1B1 promoter was increased by 1.8–3.6-fold in WY-14643-treated cells (Figure 4D), suggesting that PPARα was specifically recruited by WY-14643 and bound to the CYP1B1 promoter. Our results suggest that WY-14643 upregulates CYP1B1 expression via promoter activation at the PPRE site. We also identified putative PPREs (−833/−813) located within the promoter region of CYP1B1.

### 2.5. Effect of Activation of PPARα and RXR by WY-14643 on Induction of CYP1B1 Expression

**Figure 5:**
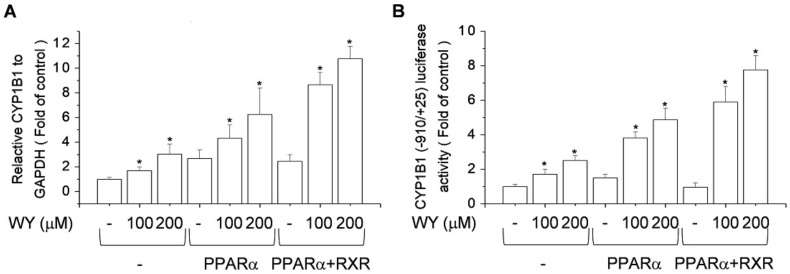
RXR and PPARα enhance the WY-14643-induced increase in CYP1B1 expression in MCF-7 cells. (**A**) Effect of co-transfection of PPARα and RXR on the WY-14643-induced increase in CYP1B1 mRNA levels. MCF-7 cells transfected with PPARα and RXR were treated with WY-14643 (100–200 μM) for 24 h. Cells were lysed and total RNA was prepared for PCR analysis of CYP1B1 mRNA levels, relative to the level of GAPDH. (**B**) Effect of co-transfection of PPARα and RXR on WY-14643-induced CYP1B1 (−910/+25) promoter activity. MCF-7 cells were transfected with CYP1B1-Luc, PPARα, or RXR, and treated with WY-14643 (100–200 μM) for 24 h. Cells were harvested and assayed for luciferase activity. Bars are means ± standard deviations of three independent experiments performed in triplicate. * *p* < 0.01, significantly different from the control.

PPAR-mediated transcription occurs primarily following heterodimerization with RXR [28]. Subsequent binding to PPREs in the regulatory regions of target genes leads to transcriptional activation. To assess whether RXR influences WY-14643-induced CYP1B1 expression, the effects of WY-14643 were compared in PPARα-expressing MCF-7 cells transfected with or without an RXR expression vector. PPARα and RXR co-transfection stimulated the WY-14643-mediated upregulation of gene expression (Figure 5A) and the reporter activity of the −910/+25 CYP1B1 promoter construct (Figure 5B). Therefore, WY-14643-induced CYP1B1 transcription was regulated by PPARα/RXR heterodimers.

## 3. Discussion

In this study, we investigated the effect of the PPARα agonist, WY-14643, on CYP1B1 expression and the related mechanism in breast cancer cells. We showed that WY-14643-induced expression of CYP1B1 was mediated by ligand-independent activation of the PPARα pathway.

The roles of PPARs have been investigated in cancer and fatty-acid metabolism, as well as in various CYP1B1-related tumorigenic states. CYP1B1 is expressed in the liver and extrahepatic tissues and mediates the metabolism of numerous xenobiotics, steroid hormones, fatty acids, and vitamins [29]. CYP1B1 is overexpressed in various types of human cancers, but not in healthy tissues [30]. Estrogen is responsible for the development and regulation of the female reproductive system, and its metabolism is associated with female cancers [29]. Estrogen, the primary sex hormone in females, is converted to 17β-estradiol by 17β-hydroxylases, such as CYP1B1 and members of the CYP1A family [31]. CYP1B1 metabolizes E2 via its primary hydroxylase activity at 2-hydroxyestradiol [32] and 4-hydroxyestradiol. The 4-hydroxyestradiol form is reportedly carcinogenic in animal models [33]. CYP1B1 is also a marker for the prevention of certain cancers, such as breast cancer [4,5]. In addition to its involvement in the activation of polycyclic aromatic hydrocarbons such as benzo[a]pyrene, CYP1B1 selectively 4-hydroxylates E2, whereas CYP1A1 and CYP1A2 mediate its 2-hydroxylation [31,34]. Because expression of CYP1B1 is elevated in various cancers, but not in normal tissues, it is a potential therapeutic target. In the present study, WY-14643 strongly induced transcriptional activation of CYP1B1 in MCF-7 breast cancer cells.

PPARs have been a focus of research that aims to identify pathways important in carcinogenesis. PPARα receptors play an important, but pleiotropic, role in malignancy; they function as tumor suppressors or promoters in a context-dependent manner. Indeed, their functions appear to be related to the type of cancer and/or tumor microenvironment. PPARα has been linked to several types of cancer, including breast cancer [16], hepatocellular carcinoma [17], chronic lymphocytic leukemia [18], glioblastoma [19,20], and renal cancer [21]. Exposure to the PPARα agonist WY-14643 caused marked upregulation of CYP1A1 in human colic adenocarcinoma-2 cells [35]. Moreover, PPARα-deficient mice were refractory to the liver carcinogenic effect of the PPARα agonist WY-14643 [22]. In addition, the growth and progression of lung carcinoma and melanoma engrafted in wild-type mice were completely suppressed when these tumors were implanted in PPARα-deficient mice [23]. Therefore, PPARα is a promising therapeutic target for cancer. The PPARα agonist, WY-14643, has proinflammatory activity, and some PPARα agonists have been shown to promote the proliferation of breast cancer cells [16,25,36]. In addition, the expression of CYP1A1 is highly induced by WY-14643 in a manner involving PPARα, which requires two PPRE sites in the CYP1A1 promoter [35]. However, the role of PPARα in the regulation of CYP1B1 expression in breast cancer cells has been unclear. We hypothesized that the WY-14643-mediated increase in CYP1B1 expression may be related to PPARα in MCF-7 cells.

Inhibition of PPARα signaling by GW6491 significantly suppressed the WY-14643-induced increase in the CYP1B1 protein level in MCF-7 cells; this was significantly suppressed by PPARα siRNA. Therefore, we concluded that WY-14643 induces CYP1B1 expression via a PPARα-dependent mechanism in MCF-7 cells. XRE, ERE, activator protein 1, and SP-1 transcription factor-binding sites are present in the CYP1B1 promoter [7]. To gain further insight into the mechanism by which WY-14643 induces CYP1B1 expression, we searched for PPARα-binding sites in the human CYP1B1 promoter. Computational sequence analysis revealed a novel putative PPARα-binding site at −942 to −772, relative to the transcription start site. Treatment with WY-14643 significantly increased the luciferase activity of PPRE. In addition, a ChIP assay confirmed that WY-14643 upregulates CYP1B1 expression via promoter activation at the PPRE-binding site. Because CYP1B1 is mainly regulated by ERE in breast cancer, we assumed that WY-14643 directly or indirectly affects this transcription factor. However, using the pGL3-CYP1B1 mPPRE (−812/+25) construct, we showed that WY-14643 failed to activate the ERE and activator protein 1 sites. Therefore, putative PPRE sites in the promoter at positions −833/−813 (CYP1B1-PPRE) are needed for the induction of CYP1B1 expression by PPARα ligands. PPARα exerts its transcriptional activity as a heterodimer with RXR. Cotransfection of PPARα and RXR stimulated WY-14643-mediated CYP1B1 expression. Therefore, WY-14643 induced-CYP1B1 transcription was regulated by PPARα/RXR heterodimers.

A recent research showed that PPARα activation by WY-14643 transcriptionally induces AhR expression through a PPRE site located within its promoter [37]. In addition, CYP1A1 and CYP1B1 were known to be expressed and induced by the transcription factor AhR. Therefore, it is possible that the increase of AhR activity may be involved in the increase of CYP1B1 expression by WY14643. The present study demonstrated for the first time that CYP1B1 was highly induced by PPARα via PPRE sites within the promoter, which indicate another strong CYP1B1 induction pathway apart from AhR-dependent pathway. Nevertheless, further studies are still needed to clearly determine how PPAR regulates the transcription of CYP1B1.

In conclusion, WY-14643 increased the CYP1B1 protein and mRNA levels, as well as promoter activity, in MCF-7 cells. Moreover, WY-14643 plus a PPARα antagonist significantly inhibited the WY-14643-mediated expression of CYP1B1. siRNA knockdown of PPARα blocked the induction of CYP1B1 expression by WY-14643, suggesting that WY-14643 induces expression of CYP1B1 via a PPARα-dependent mechanism. We identified putative PPREs (−833/−813) in the promoter region of the CYP1B1 gene; their inactivation by deletion mutagenesis suppressed the induction of CYP1B1 promoter activation by WY-14643. Furthermore, ChIP assay results revealed that WY-14643 induced activation of PPARα to a form capable of binding specifically to the PPRE sequence of the CYP1B1 promoter. In conclusion, we found that WY-14643, a PPARα agonist, increased CYP1B1 expression and activity by activating PPARα.

## 4. Materials and Methods

### 4.1. Chemicals and Reagents

WY-14643, GW6471, fenofibrate, and 2,3,7,8-tetrachlorodibenzo-*p*-dioxin (TCDD) were purchased from Sigma Chemicals Co. (St Louis, MO, USA). Dulbecco’s modified Eagle’s medium, fetal bovine serum, and penicillin-streptomycin solution were purchased from Welgene (Gyeongsan, Republic of Korea). Lipofectamine™ 2000 transfection reagent was purchased from Invitrogen (Carlsbad, CA, USA). Enhanced chemiluminescence solution was purchased from Biofact (Daejeon, Republic of Korea). Nitrocellulose membrane was purchased from Amersham Pharmacia Biotech (Piscataway, NJ, USA). A luciferase assay system was obtained from Promega (Madison, WI, USA). pCMV-β-galactosidase was purchased from TaKaRa Bio (Kusatsu, Japan). Oligonucleotide polymerase chain reaction (PCR) primers were custom synthesized by Bioneer (Seoul, Republic of Korea). Antibodies against β-actin, CYP1B1, and PPARα were from Santa Cruz Biotechnology (Santa Cruz, CA, USA), and horseradish peroxidase-linked anti-rabbit and anti-mouse secondary IgGs were purchased from Cell Signaling Technology (Beverly, MA, USA). A 3-(4,5-dimethylthiazol-2-yl)- 2,5-diphenyltetrazolium bromide (MTT) assay kit was purchased from Abcam (Cambridge, MA, USA), and a lactate dehydrogenase cytotoxicity detection kit was purchased from Roche (Mannheim, Germany). All chemicals were of the highest commercially available grade.

### 4.2. Cell Culture and Treatment

Human breast cancer MCF-7 and MDA-MB-231 cells were obtained from the American Type Culture Collection (Rockville, MD, USA). The cells were cultured to 70–80% confluence in a humidified 5% CO_2_ incubator at 37 °C in Dulbecco’s modified Eagle’s medium supplemented with 10% heat-inactivated fetal bovine serum. WY-14643, GW6471, fenofibrate, and TCDD were prepared in dimethylsulfoxide and the working concentrations were added directly to the culture medium. The final dimethylsulfoxide concentration did not exceed 0.1%, and the solvent had no noticeable effect on the assays.

### 4.3. Measurement of Cell Viability and Cytotoxicity

MCF-7 cells were seeded at a density of 4 × 10^4^ cells per 500 µL in 48-well plates. After incubation for 24 h, the growth medium was replaced with serum-free medium and the cells were treated with WY-14643 (30–300 µM) or an equal volume of dimethylsulfoxide for 24 h at 37 °C. Culture supernatants were subjected to analysis using the lactate dehydrogenase assay and the absorbance at 490 nm was measured using a microplate reader (Varioskan; Thermo Electron, Waltham, MA, USA). Cell viability was determined using the MTT assay. The cells were treated with MTT solution (final concentration, 0.5 mg/mL) for 1 h; the dark blue formazan crystals that formed in intact cells were solubilized with dimethylsulfoxide, and the absorbance at 570 nm was measured using a microplate reader. Relative percentages of cell viability and cytotoxicity were calculated based on absorbance values relative to those of the control.

### 4.4. Plasmids

The human CYP1B1-Luc vector (−1635 to +588) was a gift from Dr. Robert Barouki [38]. The PPARα expression vector was purchased from OriGene Technologies (Rockville, MD, USA). Human CYP1B1-Luc deletion plasmids were constructed to test for promoter activity using a luciferase reporter assay system. Two DNA fragments, −910 to +25 and −812 to +25, containing CYP1B1 promoter regions were amplified by PCR using the following primers (5′→3′: CYP1B1-5′-*Bgl*II, GAA GAT CTG CCC TAA GAA CTC CAG GCT TC; CYP1B1-3′-*Bgl*II, GAA GAT CTG GGG ACA GAG AGG AGA AGG CG; CYP1B1-5′-*Kpn*I, GGG GTA CCG CCC TAA GAA CTC CAG GCT TC; and CYP1B1-3′-*Hind*III, CCC AAG CTT CTG GAG TCG CAG AAG CGC TCC. All PCR products were sequenced and confirmed to be identical to the published sequence of the CYP1B1 promoter.

### 4.5. Transfection and Luciferase Assays

Using Lipofectamine 2000 reagent, cells were transiently transfected with 1 μg of hCYP1B1-Luc, an hCYP1B1-Luc deletion plasmid containing the promoter regions (−910/+25), 0.5 μg of pPPARα and RXR, and 1 μg of PPRE-Luc vector and/or 0.2 μg of pCMV-β-gal plasmid. At 5 h after transfection, fresh medium was added. The cells were treated with WY-14643 or TCDD for 24 h, then lysed. The luciferase and β-galactosidase activities in cellular extracts were measured as described previously [39,40]. Luciferase activity was normalized to the activity of β-galactosidase and expressed relative to the activity of the control group.

### 4.6. Western Blotting

After treatment, MCF-7 cells were lysed in lysis buffer (120 mM NaCl, 40 mM Tris [pH 8.0], and 0.1% nonidet P-40) on ice for 30 min and centrifuged at 12,000 rpm for 20 min. Supernatants were collected and protein concentrations were measured using a protein assay kit (Pro-Measure, Intron Biotechnology, Seongnam, Korea). Aliquots of the lysates (50 µg protein) were boiled for 5 min and resolved by using 10% sodium dodecyl sulfate–polyacrylamide gel electrophoresis. The proteins were transferred to polyvinylidene difluoride membranes and incubated with the appropriate primary antibodies. The membranes were incubated with the secondary anti-mouse or anti-rabbit antibody. Finally, the protein bands were detected using an enhanced chemiluminescence western blotting detection kit (Biofact). To investigate multiple protein targets under the same treatment conditions, the blot was stripped and re-probed. Equal sample loading was confirmed by measuring β-actin levels.

### 4.7. Quantitative Real-Time Reverse Transcription-PCR

After treatment with WY-14643 (100–200 µM) or fenofibrate (25–100 µM), MCF-7 cells were subjected to total RNA isolation using the RNAiso Plus Reagent (Takara, Tokyo, Japan), in accordance with the manufacturer’s instructions. The concentration and purity of extracted RNA were determined by measuring absorption with the NanoDrop system (ND-1000 Spectrophotometer; Thermo Scientific, Wilmington, DE, USA). cDNA was synthesized with the ImProm-II™ Reverse Transcriptase system (Promega, Madison, WI, USA). Product formation during PCR was monitored continuously using Sequence Detection System software (ver. 1.7; Applied Biosystems, Foster City, CA, USA). PCR products were detected directly by monitoring increases in the reporter dye (SYBR1) signal. The mRNA levels of CYP1B1 and PPARα in exposed cells were compared with those in control cells at each time point using the comparative cycle threshold (Ct) method (Johnson et al., 2000). The following primers were used: human CYP1B1 forward, 5′-TTC GGC CAC TAC TCG GAG C-3′; human CYP1B1 reverse, 5′-AAG AAG TTG CGC ATC ATG CT-3′; human PPARα forward, 5′-GCT GGT GCA GAT CAT CAA GAA G-3′; human PPARα reverse, 5′-GGT GTG GCT GAT CTG AAG GAA-3′; human β-actin forward, 5′-TGG CAC CCA GCA CAA TGA A-3′; human β-actin reverse, 5′-CTA AGT CAT AGT CCG CCT AGA AGC A-3′. The quantity of each transcript was calculated as described in the instrument manual and was normalized to the amount of β-actin as a housekeeping gene.

### 4.8. siRNA Transfection

Small interfering RNAs (siRNAs) targeting PPARα and ERα and the siRNA transfection reagent were obtained from Santa Cruz Biotechnology. MCF-7 cells were grown to 50% confluence and transfected for 48 h with the PPARα siRNA or a nonspecific control siRNA, in accordance with the manufacturer’s instructions. MCF-7 cells were grown to 50% confluence and transfected for 24 h with the ERα siRNA or a nonspecific control siRNA, in accordance with the manufacturer’s instructions; the cells were then treated with WY-14643 for a further 24 h.

### 4.9. Chromatin Immunoprecipitation Assay

MCF-7 cells were treated with WY-14643 for 24 h and subjected to chromatin immunoprecipitation (ChIP) assay using a ChIP assay kit (Upstate, Lake Placid, NY, USA). Anti-PPARα antibodies or normal rabbit IgG (control) were used for immunoprecipitation of protein–DNA complexes. The forward and reverse primers for CYP1B1-PPRE were 5′-GTG TCA GGT GCC GTG AGA A-3′ and 5′-GCG AAC TTT ATC GGG TTG AA-3′, respectively. PCR analyses were performed as follows: after initial denaturation at 94 °C for 3 min, amplification was performed by denaturation at 94 °C for 30 s, annealing at 58 °C for 30 s, and extension at 72 °C for 30 s for 35 cycles. The PCR product was electrophoresed on a 2% agarose gel and visualized by SYBR Green staining.

### 4.10. Statistical Analysis

All experiments were performed at least three times, and the reported values are means of three independent experiments, each performed in triplicate. One-way analysis of variance was conducted to compare differences between treatment groups. *p*-values < 0.01 were considered to indicate statistical significance.

## Supplementary Materials

Supplementary materials can be found at https://www.mdpi.com/1422-0067/20/23/5928/s1. Figure S1: Effect of WY-14643 on CYP1B1 mRNA levels in MDA-MB-231 cells, Figure S2: Effect of fenofibrate on CYP1B1 mRNA levels in MCF-7 cells

## Abbreviations

AhR	Aryl hydrocarbon receptor
CYPs	Cytochrome P450
CYP1B1	Cytochrome P450 1B1
E2	17 β-Estradiol
MTT	3-(4,5-Dimethylthiazol-2-yl)- 2,5-diphenyltetrazolium bromide
4-OHE2	4-Hydroxyestradiol
PCR	Polymerase chain reaction
PPARα	Peroxisome proliferator-activated receptor α
PPRE	Peroxisome proliferator response element
RXR	Retinoid X receptor
TCDD	2,3,7,8-Tetrachlorodibenzo-*p*-dioxin
