# Supplementary material for: WY-14643 Regulates CYP1B1 Expression through Peroxisome Proliferator-Activated Receptor α-Mediated Signaling in Human Breast Cancer Cells
Source: Int J Mol Sci. 2019 Nov 25;20(23):5928. doi: 10.3390/ijms20235928 (PMC6928855; doi:10.3390/ijms20235928)
Supplement: Supplementary file 1 [file ijms-20-05928-s001.pdf]

Supplementary figure

Figure S1

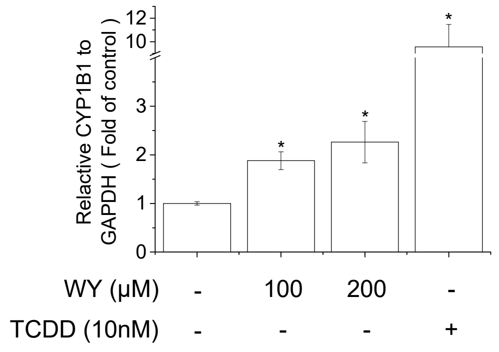

Figure S1. Effect of WY-14643 on CYP1B1 mRNA levels in MDA-MB-231 cells. Cells were treated with WY-14643 (100 and 200 μM) or TCDD (10 nM) for 24 h, then lysed; total RNA was prepared for PCR analysis of CYP1B1 mRNA levels, relative to the level of GAPDH. Bars are means ± standard deviations of three independent experiments performed in triplicate. *$p < 0.01$, significantly different from the control.

Figure S2

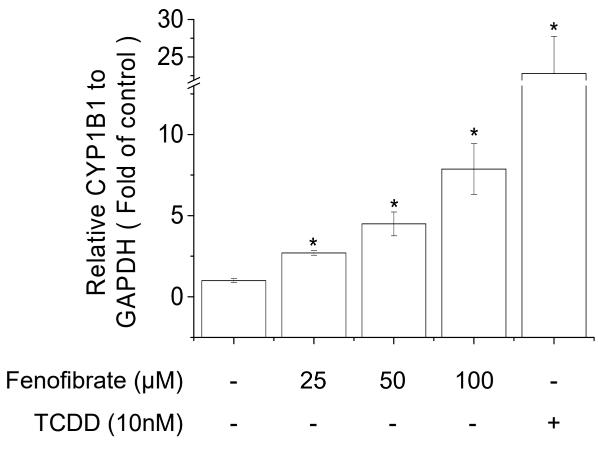

Figure S2. Effect of fenofibrate on CYP1B1 mRNA levels in MCF-7 cells. Cells were treated with fenofibrate (25, 50, and 100 μM) or TCDD (10 nM) for 24 h, then lysed; total RNA was prepared for PCR analysis of CYP1B1 mRNA levels, relative to the level of GAPDH. Bars are means ± standard deviations of three independent experiments performed in triplicate. *$p < 0.01$, significantly different from the control.
